# Spontaneous rupture of renal angiomyolipoma with characteristic skin findings

**DOI:** 10.1016/j.jdcr.2025.10.055

**Published:** 2025-11-06

**Authors:** Hoang Quoc Tuan, Nguyen Thi Quynh Trang, Nguyen Lan Anh, Tran Hien Luong, Nguyen Huong Giang

**Affiliations:** a Department of Dermato-Venereology, Military Central Hospital 108, Hanoi, Vietnam; b Department of Diagnostic Imaging, E Hospital, Hanoi, Vietnam; c Department of Dermatology, Mayo Clinic, Rochester

**Keywords:** Renal angiomyolipoma, spontaneous rupture, skin findings

## Case description

**Fig 1:**
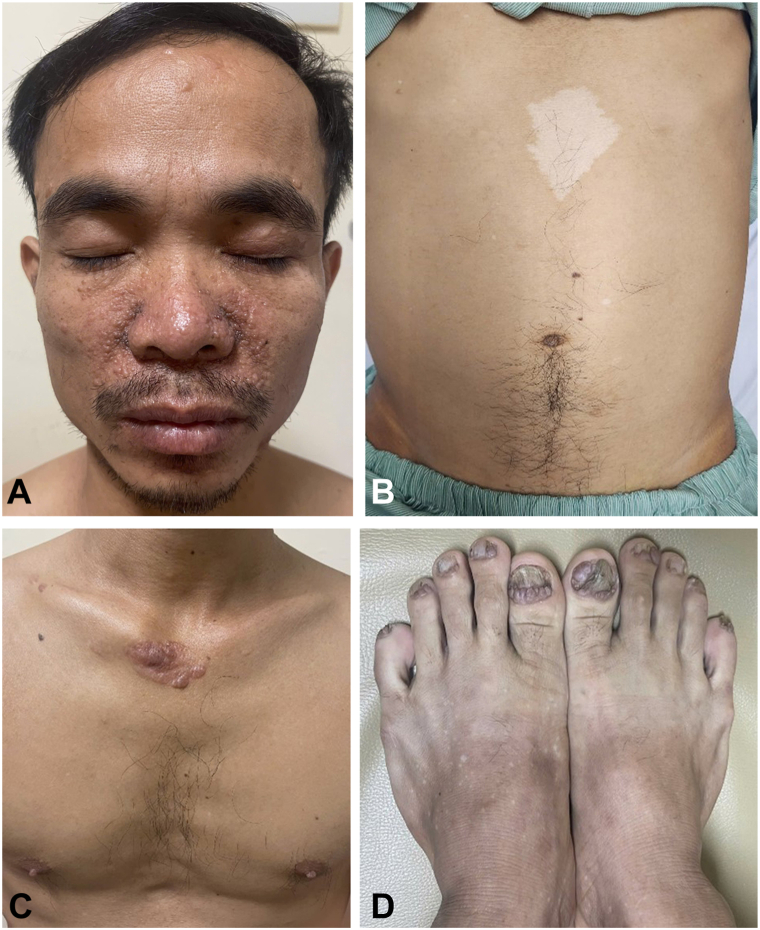
Cutaneous manifestations in the patient. **A,** Multiple facial angiofibromas. **B,** Hypopigmented ash-leaf macule on the abdomen. **C,***Shagreen* patch on the upper chest. **D,** Periungual fibromas arising from the toenail folds.

A 38-year-old man presented with a 3-day history of acute abdominal pain. He appeared pale with moderate abdominal tenderness. Dermatologic examination revealed several notable findings: multiple discrete, firm, flesh-colored to reddish-brown papules (2-4 mm) clustered over the nasolabial folds and cheeks, consistent with facial angiofibromas (Fig 1, *A*). Several hypopigmented, oval-to-lanceolate macules (1-3 cm) scattered on the trunk, consistent with ash-leaf macules (Fig 1, *B*). A raised, flesh-colored to pink plaque with a leathery texture was noted on the upper chest, suggestive of a Shagreen patch (Fig 1, *C*). Multiple periungual fibromas involving the toenails were also present, appearing as firm pink nodules arising from the nail folds (Fig 1, *D*). He reported that these lesions had been present since childhood but were never evaluated. His father and brother had similar cutaneous findings.

**Fig 2:**
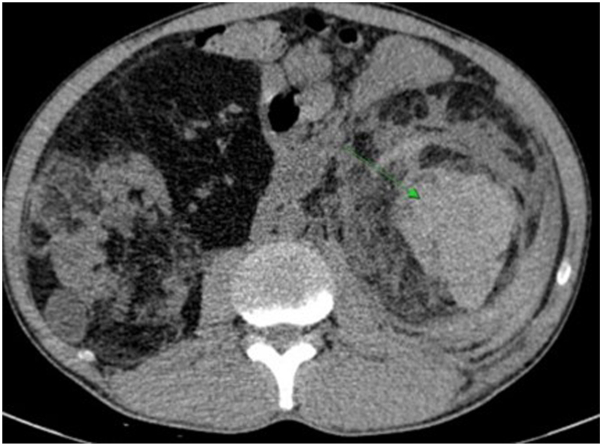
Contrast-enhanced CT of the abdomen showing a ruptured left renal angiomyolipoma (*arrow*) with a large retroperitoneal hematoma. Multiple bilateral renal AMLs are also visible.

Laboratory tests revealed normocytic anemia (hemoglobin 10.2 g/dL), leukocytosis (WBC 13.6 × 10^9^/L), elevated serum creatinine (130 μmol/L) and markedly increased C-reactive protein (CRP 220 mg/L). Contrast-enhanced computed tomography (CT) of the abdomen showed enlarged bilateral kidneys with multiple heterogeneous masses containing fat, soft tissue and vascular components. A large lesion in the lower pole of the left kidney (88 × 89 × 99 mm) demonstrated areas of high attenuation compatible with acute hemorrhage, along with perirenal and retroperitoneal hematoma, consistent with rupture of an angiomyolipoma. The right kidney contained smaller heterogeneous lesions, the largest measuring 115 × 79 mm, with several fat-density foci. These findings were consistent with bilateral renal angiomyolipomas with spontaneous rupture of the left-sided lesion (Fig 2). The constellation of renal and cutaneous findings suggested an underlying multisystem genetic disorder, which was subsequently confirmed based on established diagnostic criteria.^1^ Urgent selective arterial embolization of the ruptured angiomyolipoma was performed successfully.

**Question:What is the most likely diagnosis in a patient presenting with spontaneous renal hemorrhage, multiple facial papules and hypopigmented macules?**

**A.** Birt–Hogg–Dubé syndrome
**B.** Von Hippel–Lindau disease
**C.** Tuberous sclerosis complex
**D.** Cowden syndrome
**E**. Neurofibromatosis type 1

**Correct answer: C. Tuberous sclerosis complex.**

## Discussion

Tuberous sclerosis complex (TSC) is a rare autosomal dominant disorder caused by mutations in TSC1 or TSC2, leading to dysregulation of the mTOR pathway.^2^ Cutaneous manifestations are often the earliest and most accessible diagnostic clues, including facial angiofibromas, Shagreen patch, periungual fibromas and hypopigmented macules.

Renal angiomyolipomas (AMLs) occur in up to 80% of patients with TSC and represent a major cause of morbidity due to the risk of rupture and hemorrhage.^3^ Although hemorrhage from renal AMLs in TSC is well recognized, it is uncommon for such bleeding to serve as the initial presentation leading to diagnosis, as in our case. Sporadic AMLs are uncommon, making recognition of cutaneous findings essential for early diagnosis.

Early dermatologic recognition is crucial for timely surveillance and prevention of complications. Current guidelines recommend renal imaging every 1-3 years.^4^ Treatment options for AMLs include embolization and mTOR inhibitors such as everolimus, which can reduce tumor volume and improve cutaneous lesions.^5^

This case highlights the key role of dermatologists in identifying classic cutaneous features of TSC, which may provide the first clue to a life-threatening systemic disease.

## Conflicts of interest

None disclosed.

## Supplementary data

Video E1

Video E2
